# The Synthesis, Characterization and Applications of Polyhydroxyalkanoates (PHAs) and PHA-Based Nanoparticles

**DOI:** 10.3390/polym13193302

**Published:** 2021-09-27

**Authors:** Antony V. Samrot, Sree K. Samanvitha, N. Shobana, Emilin R. Renitta, P. Senthilkumar, Suresh S. Kumar, S. Abirami, S. Dhiva, M. Bavanilatha, P. Prakash, S. Saigeetha, Krithika S. Shree, R. Thirumurugan

**Affiliations:** 1 School of Bioscience, Faculty of Medicine, Bioscience and Nursing, MAHSA University, Jalan SP2, Bandar Saujana Putra, Jenjarom 42610, Selangor, Malaysia; 2 Department of Biotechnology, Shanmugha Arts, Science, Technology & Research Academy, Thanjavur 613401, Tamil Nadu, India; sreesamanvitha95@gmail.com; 3 Department of Biotechnology, School of Bio and Chemical Engineering, Sathyabama Institute of Science and Technology, Chennai 600119, Tamil Nadu, India; shobanan1993@gmail.com (N.S.); bavagold@gmail.com (M.B.); kpprakashmtech@gmail.com (P.P.); rajivarsha2000@gmail.com (S.S.); krithikasivasuriyan09@gmail.com (K.S.S.); 4 Department of Food Processing Technology, School of Agriculture and Biosciences, Karunya Institute of Science and Technology, Karunya Nagar, Coimbatore, 641114, Tamil Nadu, India; renitta@karunya.edu; 5 Department of Chemical Engineering, School of Bio and Chemical Engineering, Sathyabama Institute of Science and Technology, Chennai 600119, Tamil Nadu, India; sensen10@gmail.com; 6 Centre for Materials Engineering and Regenerative Medicine, Bharath Institute of Higher Education and Research, Chennai 600126, Tamil Nadu, India; 7 Department of Microbiology, Kamaraj College, Thoothukudi 628003, Tamil Nadu, India; abisasi@gmail.com; 8 Department of Microbiology, Sree Narayana College, Alathur, Palakkad 678682, Kerala, India; dhivasoju@gmail.com; 9 Department of Transfusion Medicine, Jawaharlal Institute of Postgraduate Medical Education and Research, Puducherry 605006, India; thirumuruganphd@gmail.com

**Keywords:** polyhydroxyalkanoates (PHAs), nanoparticles, extraction, synthesis, applications

## Abstract

Polyhydroxyalkanoates (PHAs) are storage granules found in bacteria that are essentially hydroxy fatty acid polyesters. PHA molecules appear in variety of structures, and amongst all types of PHAs, polyhydroxybutyrate (PHB) is used in versatile fields as it is a biodegradable, biocompatible, and ecologically safe thermoplastic. The unique physicochemical characteristics of these PHAs have made them applicable in nanotechnology, tissue engineering, and other biomedical applications. In this review, the optimization, extraction, and characterization of PHAs are described. Their production and application in nanotechnology are also portrayed in this review, and the precise and various production methods of PHA-based nanoparticles, such as emulsion solvent diffusion, nanoprecipitation, and dialysis are discussed. The characterization techniques such as UV-Vis, FTIR, SEM, Zeta Potential, and XRD are also elaborated.

## 1. Introduction

Polyhydroxyalkanoates (PHAs) are polyesters that contain a characteristic bond of esters, which are accumulated as carbon and energy reserve along with limited nitrogen source and assist in providing energy [1,2,3,4,5]. The structure of PHAs is composed of 3-hydroxy fatty acid monomers [6,7,8]. They are believed to be biodegradable and biocompatible in nature [9]. PHAs are divided into groups based on the number of carbon atoms present in the monomer units produced by them: Short Chain Length (scl-PHAs)—these contain three to five carbon atoms in a monomer; Medium Chain Length (mcl-PHAs)—these contain 6 to 14 carbon atoms in a monomer [10]; Long Chain Length (lcl-PHAs)—these contain 15 or more than 15 carbon atoms in a monomer. The molecular weight of polyhydroxyalkanoates (PHAs) cause them to have characteristics comparable to thermoplastic compounds. The durability, stability and flexibility of PHAs and conventional thermoplastics are similar [11]. The properties of Short Chain Length (scl-PHAs) are close to conventional plastics because of its physical and mechanical properties [12]. They are crystalline in nature and are usually rigid, hard structures. Medium Chain Length (mcl-PHAs) have elastomeric, adhesive properties and are amorphous thermoplastics with varying degrees of crystallinity [13].

The physical and chemical properties depends upon the monomeric composition of PHA producing microbes and its nutrition [14]. Due to the numerous structural and mechanical features possessed by PHAs, they are being applied in various fields of technology [15,16]. The most important property of PHAs is that they do not pollute the environment as conventional plastics do, which makes them an exceptional resource with properties similar to commercially available synthetic plastics. However, the major constraint with PHAs is the higher production cost [17]. Polyhydroxybutyrate (PHB) is a widely used Polyhydroxyalkanoate and is a macromolecule which consists of repeating monomers of butanoic acid. Lemoigne [18] first reported the formation of poly(3-hydroxybutyrate) (PHB) inside a bacterial cell. PHB is a principal reserve of bacteria for storing carbon and energy [19]. There are other PHAs that contain various hydroxy fatty acids such as octanoic acid, hexanoic acid, valeric acid, and their respective copolymers. Various PHAs are synthesized by providing other substrates to the microorganisms [20,21]. Yield10 Bioscience is a company involved in developing new technology in the agricultural field [19]. They have analyzed a copolymer for trading on a large scale and to overcome the limitation in the production cost. A Gram-negative soil bacterium named *Cupriavidus necator* was used along with a naturally occurring carboxylic acid and a simple sugar. Further reductions were made by using non-depleting energy sources such as activated sludge [22,23,24]. The produced copolymer possessed properties similar to the existing homopolymer and showed better industrial applications. The accumulation of PHA in *Cupriavidus necator* was almost equivalent to the dry weight of the entire biomass [20].

### 1.1. PHA Biosynthesis

**Figure 1:**
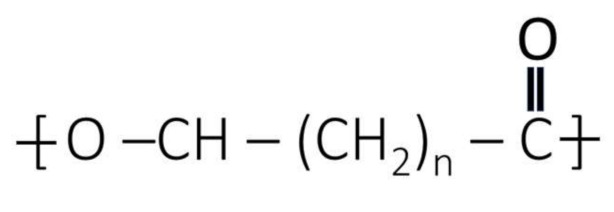
Basic structure of PHA.

**Figure 2:**
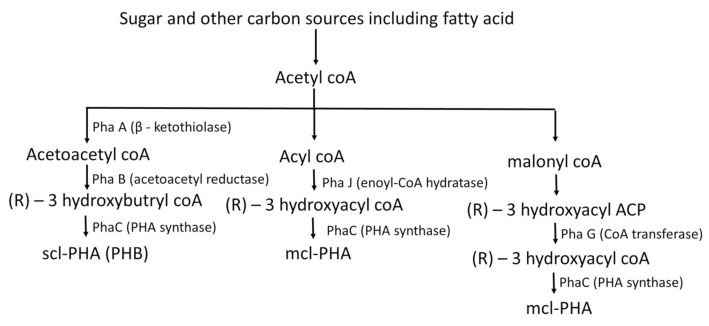
Biosynthesis of Polyhydroxyalkanoate (PHA).

PHA is widely produced by microorganisms irrespective of whether it is a Gram-positive or Gram-negative bacterium [25]. Various microorganisms synthesize polyhydroxyalkanoates (PHAs) as a physiological strategy related to the utilisation of nutritional resources in an ecosystem [26]. PHAs can be accumulated in about 94% (*w/w*) of the dry cell mass inside the bacterial cells [2] and the basic structure is illustrated in Figure 1. They are produced continuously through a chain of chemical conversions that occur in the cell and PHA is accumulated within the cytoplasm of the cell as energy reserves. PHAs appear to be similar to a compact granule inside the cell and provide the nourishment to the cell for growth when surplus carbon is provided [27]. The granules of the PHAs are coated with phospholipids and proteins called phasins, and these phasins play a vital role in size of the PHA granule [28,29]. The production pathway of PHAs starts as a sequence wherein *β*-ketothiolase effectuates the formation of two acetyl-CoA molecules. The acetyl CoA molecule sustains a NADPH-dependent reaction that catalyzes the addition of hydrogen to impart hydroxybutyryl CoA molecules. The PHB enzyme synthase converts hydroxybutyryl CoA molecules into PHB granules [30]. The yield of PHA is directly proportional to the correspondence of the co-enzymes NADH and NAD+ [31] which restrict acetyl-CoA entering the Krebs cycle [32] (Figure 2). Sometimes, mcl-PHA is synthesized from longer carbon chain monomers wherein the β-oxidation of long chain fatty acids converts into monomers of (R)-3-hydroxyacyl-CoA which takes place through the action of an enzyme named enoyl-CoA hydratase (PhaJ) [33], where the mcl-PHA is synthesized by PhaC (PHA synthase). From the simple sugars, fatty acids are synthesized through a *de novo* pathway leading to the formation of 3-hydroxyacyl (3HA) precursors. These precursors are converted into acetyl-CoA and then to malonyl-coA, where specific CoA transferase (PhaG) forms (R)-3-hydroxyfatty acids from malonyl coA [34,35]. (R)-3-hydroxyfatty acids are accumulated in the form of mcl-PHA by Pha C [36,37,38].

### 1.2. Properties of PHA

The chemical and physical properties of PHAs vary considerably with the monomer composition. PHAs are insoluble in water [39]. Generally, polymers are characterized using parameters such as hydrophobicity, melting point, glass transition temperature, and degree of crystallinity. The family of PHAs exhibits a wide variety of mechanical properties from hard crystalline to elastic [40]. Biodegradability is one of the most distinctive properties of PHA. It is naturally extracted from microorganisms and can be broken down into water and CO_2_ in seven months if buried in soil [41]. Multitudinous microorganisms existing in nature discharge PHA-abrading enzymes, allowing the breakdown of PHA present in the environment into supplements that dissolve in water, subsequently employing the produce as a source of nutrition. PHA is deteriorated by the effort of equivocal esterases and its subclasses. Löbler et al. [42] perceived the activity of the enzyme lipases, a subclass of esterases in the rat gastrointestinal near the PHA insert and considered the complicity of lipases in the chemical processes of PHA taking place within the organism.

The deterioration of PHA embedded in the specialized cells of the host organism of interest extends the likelihood of pairing this occurrence with the liberation of bioactive compounds, such as antibacterial, antifungal, or anticancer drugs. For instance, if a cell is saturated with a substance, the deterioration over a span of time would liberate the substance, substituting as a self-activating operative [42]. PHB is widely used as implant materials. The significance of using implants made with PHB is that the body does not consider it as a foreign object, so the immune system does not respond to nor reject the implant [43]. These impressive properties of PHB make it an ideal material for nano-entrapment and the delivery of antimicrobial compounds.

### 1.3. Production of PHA

Many research efforts are being carried out to identify an optimal mixture of mixed microbial cultures that could generate PHA constantly, without any reduction in the production rate, while using a continuous supply of a complicated substrate to keep the consortia functioning [44]. A surplus amount of carbon as a substrate accommodates depositories of PHA and diminishes the biomass production. The microorganisms that assemble PHA are expedient compared to other microorganisms that cannot amass PHA, as the accumulated PHA can be used as an instantaneous source of energy in transient conditions [45]. The yield of PHA from biomass can be increased by finding the best appropriate carbon source [46,47]. PHA accumulation was investigated in *Enterobacter cloacae* SU-1 isolated from the soil of *Arachis hypogea*. In the lactose-containing media, 94% of the dry weight of PHA was accumulated [2]. Beun et al. [48] made a concerted effort to understand the PHA produced by the biological floc in the process of converting a carbon supply, and eventually concluded that a fluctuating carbon source leads to the disordered production of PHA. The biological floc of microorganisms employed in the production of PHA exhibited a decreased yield of PHA when compared to the yield of PHA from an individual organism. Satoh et al. [49] conducted a comprehensive analysis of PHA production from biological flocs and found that by focusing on its optimization, the level of PHA production from activated sludge may be elevated. An effective procedure can be completed by paying attention to the combination of the parameters used in the production of PHA. Lee et al. [50] found the change of propionate and hydroxyvalerate content in the polymer chain of PHA when the glucose concentration was changed. Feasible production of microbial PHA in bulk quantity it depends upon four magic “e” namely: ethical, environmental, engineering and economic aspects [51]. The production of PHA from various microorganisms is listed in Table 1.

## 2. Optimization of PHA Production Using Response Surface Methodology

The conventional method to optimize a multivariable process requires more experimental data and is an extensive and time-consuming process [70,71]. This limitation could be solved by varying multiple variables simultaneously. Using statistical techniques such as Response Surface Methodology (RSM), the experimental runs can be designed (Design of Experiment) in a way that the number of experimental runs required for the statistical analysis are reduced [72,73]. Compared to the One-Factor-At-a-Time (OFAT) analysis, RSM helps to understand the interaction between various input variables at the same time [74]. Usually, all optimization techniques contain the following five steps [75,76]:

- Identifying the key variables which majorly influence the process output;
- Conducting the experiments which are designed using statistical tools;
- Choosing a proper mathematical model for the process and estimating its coefficients;
- Simulating the process output using the developed model;
- Verifying the tolerability of the model at various process conditions.

The contour plots, response surface, multivariate regression analysis, and interpretations of the resulting equations are carried out in the final step. There are certain assumptions in the ANOVA method such as: each sample is taken from normally distributed responses and all samples have a homogeneity of variance. So, the RSM technique can be used to develop the model and to optimize several input parameters in a shorter time compared to conventional optimization techniques [77].

The response surfaces are represented by mathematical model equations which are similar to regression equations [78]. First or second order regression equations can be used to analyze the responses of a system to various independent input parameters [79]. However, first order models may fail to generate a valid response if the relation between the process variables and the response variables are complex. In that case, second or higher order models could be used to obtain a valid result [77,80].

Previous reports by researchers have confirmed that the use of RSM can yield better optimum conditions for the increased production of PHAs by different bacterial strains [81]. Kumar et al. [82] reported a 30% increased bacterial biomass and 66% of PHA content compared to the conventional optimization method from *Pandoraea* sp. ISTKB. PHA production by *Acinetobacter junii* was 5.84 times higher than the PHA produced with the parameters identified using the “one-factor-at-a-time” optimization technique [83]. The yield of PHA by *Wickerhamomyces anomalus* VIT-NN01 was reportedly increased to 19.5 g per L from 3.7 g per L under optimized conditions using sugarcane molasses as a medium [84].

### Optimization of PHA Production by Various Parameters

The optimization of cultural conditions through experimental design approaches has recently been used to increase PHA production using a limited number of experiments among the various parameters applied for enhancing the production of PHAs. Conventional experimental approaches, in contrast to statistical methods, analyze one factor at a time while holding other factors stable, resulting in an excessive number of time-consuming experiments that do not include details about factor interaction [5,85].

In a 48-h incubation period, Gram-negative *E. cloacae* SU-1 was able to accumulate a high concentration of PHA (94 percent dry weight) in a minimal medium containing 8 g/l lactose at a pH of 7.5 and 30 °C [2]. Zihayat et al. [86] studied five different parameters such as glucose, K_2_HPO_4_ concentration, NH_4_Cl, pH, and temperature for PHA production in the *Pseudomonas pseudoalcaligenes* strain Te using a D-optimal design. Among them, pH, K_2_HPO_4_, and temperature were found to have a major impact on PHA production. *Pseudomonas aeruginosa* SU-1 produced PHA that was 1.3 times higher at a pH of 8, a temperature of 40 °C, and a 48 h incubation period [5]. The effects of a different pH, temperature, incubation time, agitation time, NaCl concentration, carbon, and nitrogen sources were considered while an optimized media for PHA production from *Bacillus megaterium* isolated from marine water was used [87]. Elsayed et al. [88] attempted to manufacture PHB from *Bacillus cereus* CCASU-P83 in a cost-effective manner. It yielded 50% PHB per dry cell weight, with a molecular weight of 26,900 g/mole.

## 3. Extraction of PHA

PHAs are produced intracellularly in the form of granules surrounded by a large number of proteins, and to obtain them in pure form, they must be extracted from the cell by breaking the cellular matrix [4]. Bacteria are treated with a mixture of enzymes that break down proteins and cell walls in the industrial setting. One of the most commonly used methods involves employing solvents or compounds that are composed of chlorine and oxygen for extracting the PHA in the best possible manner, or by implementing the service of enzymes that digest the negated cellular material other than PHA [52,89,90]. Holmes et al. [91] shed light on a possible alternative for the improvement of PHA extraction that includes the treatment of the biomass with a specific catalyst resulting in a biochemical reaction alongside an anionic detergent under an optimum temperature. This technique is now being carried out at Zeneca Bio Products in the United Kingdom but is unsuited for scaling up because of the susceptibility of the number of enzymes and chemicals used in the process [92].

Fidler et al. [93] formulated a new theory that a phage containing a sequence of nucleotides can disintegrate the cell wall of *E. coli* that produces PHA due to the different combination of the alleles. This method of disintegration does not require the inclusion of solvents, compounds containing chlorine and oxygen, or the introduction of any specific catalysts. Resch et al. [94] used a sequence of nucleotides of bacteriophage that multiplies within a bacterial cell to complete a systematic study of the intracellular PHA accumulated during the inert phase of the bacterial cell. This was completed to prevent the buildup of MgSO_4_ in the cell wall, which helps the rigidity of the cell wall. In the absence of MgSO_4_, the accumulated PHA can be recovered under heat sensitive conditions. When PHA granules are exposed to biological substances or physical processes, they become denatured quickly. To circumvent this, it has been suggested that PHA granules must be retrieved using mechanical or enzymatic cell lysis followed by centrifugation for recovery. The form and structure of PHA granules, as well as their shell-core composition, may be preserved in this fashion [95].

### 3.1. Solvent Extraction

Solvent extraction is the most widely used method to extract PHA from the microbial cell mass because the technique is simple, efficient, fast, and gives PHA with a high purity and molecular weight [96]. The microbial cells are made permeable to allow the PHA granules to solubilize and release out of the cell followed by the precipitation by solvents. Various solvents such as chloroform, ethanol, methanol, hexane, sodium hypochlorite, 1,1,2-dichloroethane, ethylene carbonate, 1,2-propylene carbonate, and acetic anhydride have been reported for the process of extraction of PHA [97]. Solvents such as acetone have been especially utilized for the extraction of mcl-PHA [98]. Jiang et al. [52] reported the use of non-chlorinated solvents such as cyclohexanone and *γ*-butyrolactone to extract PHA from *Cupriavidus necator* H16 where vegetable oil was used as the carbon source. The use of alkalis, such as NaOH or KOH for PHA extraction is regarded to be one of the cheapest and lower cost techniques [99].

### 3.2. Digestion Method

The digestion method is an alternative method for extracting PHA from the cell biomass as the solvent method involves the usage of various solvents which can be harmful and toxic to the environment when used on a large scale. When a cell is lysed, the membrane-bound PHA granules are loosened, and the hydrophilic non-PHA cell mass is altered to form a water-soluble material with the help of chemical or enzymatic activity [100]. It can be of two types: Chemical Digestion or Enzymatic Digestion. The main principle behind Chemical Digestion is the solubilization of cell components (non-PHA cell mass) using various surfactants such as sodium dodecyl sulfate (SDS), Triton X-100, palmitoyl carnitine, betaine, or even sodium hypochlorite, which acts as a strong oxidizing agent [96]. Usually, a combination of surfactants is used for the process while a high pH is maintained [101]. To solubilize the hydrophilic substance from the cell, a concentration of 0.025–0.2% SDS is generally employed. A greater concentration of SDS cause the PHA to solubilize, lowering the yield [102]. However, because this method has a significant risk of generating hazardous halogenated chemicals, it is not widely used for extracting PHA from cells [100]. 

Enzymatic digestion involves the use of various types of proteolytic enzymes such as alcalase, lysozyme, etc which result in the dissolution of intracellular components such as proteins, leaving behind PHA granules which are then separated by simple filtration and extracted using a suitable solvent [103,104]. The use of other enzymes such as bromelain and pancreatin has also been reported. The enzymatic technique typically includes a thermal pre-treatment of the biological material, followed by enzymatic hydrolysis, which is then treated with surfactants before being decolored with H_2_O_2_ [102]. A proteolytic and caseinolytic enzyme produced from *Streptomyces albus* has been utilized to extract PHA from *B. megaterium* with a high purity of 90–93% [105].

### 3.3. Mechanical Method

**Figure 3:**
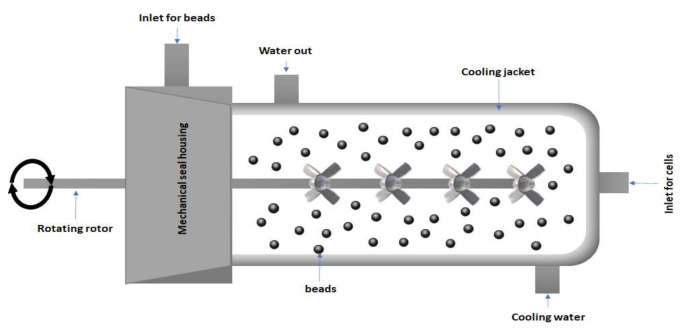
Bead Milling (Inspired from Diels and Michiels [112]).

The mechanical method is a method where the cell wall components are broken by a mechanical force and is used mostly to recover intracellular components such as PHA without the use of any solvents [106]. It is a promising method for use on an industrial scale. The principle behind bead milling or bead homogenization is based upon shearing force, impact force, hitting force, and energy transfer from the beads to the cell mass (Figure 3). Bead milling is independent of the biomass concentration [107]. Bead diameter, bead density, bead filling, agitator speed, and feed rate must be optimized depending upon the rigidity of the cell. It consists of a grinding cylinder with variable speed rotor for grinding. The grinding process generates heat which is counter balanced by circulating cooling water surrounding the chamber. Although the alkaline pretreatment of cells containing PHA granules enhances single-pass disruption in a high-pressure homogenizer, full protein release requires at least two passes [108]. To improve single-pass disruption, substances such as 0.1% SDS, sodium chloride, potassium chloride, lysozyme, EDTA, or mixtures of these can be employed [109]. Gutt et al. [110] used ANOVA models to improve a technique for extracting P3HBHV from *Cupriavidus necator*. They devised a technique that combines mechanical disintegration with surfactant treatment, which has been shown to be more effective than utilizing only one. The intricacy of the parameter interactions makes the bead milling process difficult to anticipate and so it is not widely used. This procedure also has a long processing time and usually requires several steps so it can be coupled with enzymatic digestion [111].

High pressure homogenization is another mechanical treatment which consists of an air-driven positive displacement pump that pushes the cell slurry under high pressure through two parallel slots [113]. The efficiency of bead milling is independent of the biomass concentration, which is opposite to the performance of high-pressure homogenization which is totally dependent on the biomass concentration [114]. When compared to Gram-negative bacteria, Gram-positive bacteria are more difficult to disrupt [112]. To enhance the quality and quantity of PHA, the mechanical disruption of cells has been supplemented with the application of solvents, surfactants, or chemicals [115].

### 3.4. Supercritical (SC) Fluids

Supercritical (SC) fluids have a low viscosity and have high densities, and thus can be used to disrupt the bacterial cells. Carbon dioxide at controlled conditions of 74 bar and 31 °C diffuses through the cells and solubilizes the PHA [116]. It is used extensively as it is environmentally friendly, rapid, and less expensive; thus, it is used on an industrial scale to recover PHAs especially for biomedical applications. In relation to this, ammonia and methanol have also been reported [117]. Using supercritical CO_2_, 89% of P(3HB) and 42.4% of mcl-PHA were recovered from *R. eutropha* and *P. resinovorans* strains, respectively. However, setting up this approach on an industrial scale is challenging due to the high maintenance costs of the equipment [118,119].

## 4. Characterization of PHA

To successfully produce PHA in a fermentation process, identification, and characterization of PHA producing microorganism using a rapid, simple, and reliable method is essential. A variety of methods are available for screening the PHA-producing bacteria, and the characterization of PHAs which can be categorized into staining, Spectroscopy, Chromatography, and Microscopy methods [120], which are discussed below.

### 4.1. Microscopy

#### 4.1.1. Staining and Microscopy

Staining using Sudan black B [121], Nile Blue A [122], or Nile Red [123,124] are traditional methods for the identification of PHA-producing bacteria. Sudan black B does not only stain PHA, but it also stains other lipid substances and inclusion bodies. For PHA detection, Nile blue A and Nile red are said to be more specific and superior compared to Sudan black B [125,126,127,128]. Sudan Black B staining in *Bacillus megaterium* SF4 revealed blue–black intracellular inclusions of PHA [129]. Wei et al. [130] also reported the use of the Sudan Black B staining method to identify the indigenous strain *Cupriavidus taiwanensis* 184 for PHB production. Kitamura and Doi [131] developed an efficient method to detect PHA-producing bacterial colonies on an agar plate. PHA granules can be stained and viewed under a phase contrast microscope. Phase contrast microscopy is frequently used to identify PHA-producing bacteria. The presence of intracellular granules confirms the existence of PHB when examined using phase contrast microscopy. This approach can be used to check many isolates in a small amount of time [132]. 

#### 4.1.2. Transmission Electron Microscopy (TEM)

Transmission Electron Microscopy (TEM) analysis has made it easy to study the structure of cells, tissues, cell organelles, etc. The sample needs to be prepared before viewing under TEM. The PHA-producing cell is manually fixed in a fixative solution containing glutaraldehyde, freshly made paraformaldehyde, and sucrose in a sodium cacodylate buffer and is fixed twice using an osmium solution and three times using a Kellenberger uranyl acetate solution. After that, it is dehydrated in an ethanol solution before being embedded in resin and hardened. It is then sectioned for viewing under TEM [133]. Berlanga et al. [134] proposed a simple method for the visualization of PHA granules under TEM. The PHA-producing cells are collected as pellets after ultracentrifugation and are fixed using glutaraldehyde and then stained with osmium tetroxide (OsO_4_) and uranyl acetate. Danis et al. [135] reported TEM images of spherical-, ovoidand elongated-shaped PHA granules at or near the cytoplasmic membranes with clear boundaries from the halophilic archaea. PHA granules were visualized within the cells of *Cupriavidus* sp. ISTL7 using TEM [136]. TEM was also utilized by Boonyawanich et al. [137] to examine the physical appearance of intracellular PHAs inside *Pseudomonas putida* TISTR 1522. It was accrued in the form of granules, with 3–10 granules per cell. These granules were white and roundish in form, with diameters of 0.3–0.5 µm. As with all staining techniques, TEM can confirm if there is an accumulation, but it does not confirm the composition. The use of expensive chemicals and sample preparation time are the drawbacks. 

#### 4.1.3. Scanning Electron Microscopy (SEM)

Scanning Electron Microscopy (SEM) can be used to study the morphology of the PHA inside the cell as it provides high resolution three-dimensional (3D) images without the need for complicated sample preparation. It can be coupled with an energy-dispersive spectroscopy (EDS) detector to provide an elemental analysis of the PHA and has been used extensively in the field of polymer sciences [138]. The sample preparation is quite easy which usually requires the nonconductive samples to be ion sputtered or chemically etched with a thin layer of metal such as gold or platinum. Using a scanning electron microscope and energy dispersive spectroscopy, the microstructure, surface morphology, and chemical composition of PHA generated by *Rhodococcus* sp. and *Lactobacillus* sp. were investigated. They were discovered to be porous materials with tiny grains linked in a regular pattern [139]. SEM pictures of poly(3-hydroxybutyrate) [P(3HB)] homopolymer produced by *Bacillus megaterium* UMTKB-1 and poly(3-hydroxybutyrate-co-3-hydroxyvalerate) P(3HB-co-3HV) copolymer produced by *Massilia haematophila* UMTKB-2 showed that the microbeads were between 8 and 56 m in size [140].

### 4.2. Spectroscopy

#### 4.2.1. UV-Visible Spectroscopy (UV-Vis)

The gravimetric method of PHA extraction is inexact and time-consuming which has led to the development of a more sensitive method by Williamson and Wilkinson [141]. The PHA-producing cells are digested in an alkaline sodium hypochlorite solution to extract the PHA granules which is followed by centrifugation to collect the granules. The extracted PHA granules are precipitated with a suitable solvent and the addition of sulfuric acid converts it into crotonic acid which can be measured by a UV Spectrophotometer. The existence of crotonic acid, which is produced via the reaction of PHA with concentrated sulfuric acid, can be read and verified by a peak between 220 and 240 nm [5]. A similar protocol was followed by Duvigneau et al. [142] to measure the PHA content in poly(3-hydroxybutyrate-co-3-hydroxyvalerate). Another simple method where PHB molecules are made to digest 2-butenoic acid by heating in the presence of concentrated sulfuric acid, and the subsequent spectrophotometric determination of polyester content at absorption band λ = 235 nm was suggested by Law and Slepecky [143]. 

#### 4.2.2. Fourier Transform-Infrared Spectroscopy (FT-IR)

Fourier-transform infrared spectroscopy (FT-IR) has proven to be a rapid and powerful tool for studying microorganisms and their cell components [144]. It provides details about the chemical structure of the PHA and its monomeric units. This method involves minimal sample preparation and can be widely used to detect the different functional groups of the PHA molecule and to distinguish between different types of PHAs due to its strong carbonyl absorption. The presence of strong carbonyl marker bands in the 1700 cm^−1^ range suggests the possible accumulation of PHA in the bacterial cells. This is due to the carbonyl group’s oxygen atoms being closer to hydrogen atoms, resulting in hydrogen–bond interactions. Bands near 1280 cm^−1^ and 1160 cm^−1^ help to identify the type of PHAs [145]. Bhuwal et al. [146] reported PHA production and identification by two bacterial isolates which used cardboard industry wastewater as sole carbon source. The PHAs produced by them had intense absorption bands around 1720 cm^−1^.

Intracellular scl-PHA, mcl-PHA, and scl-mcl-PHA have a characteristic ester carbonyl band at 1732 cm^−1^, 1744 cm^−1^, and 1739 cm^−1^, respectively, whereas pure polymers scl-PHA, mcl-PHA, and scl-mcl-PHA had the same band at 1728 cm^−1^, 1740 cm^−1^, and 1732 cm^−1^, respectively [147]. The presence of a short chain length PHB was confirmed by Tyagi and Sharma [148] in *Ancylobacter* sp. isolated from crude paper industry effluent. Bands at 1720, 1452.40, and 1276 cm^−1^ corresponding to PHB were found in the spectrum. FTIR spectroscopy has also proven to be a useful technique for determining crystallinity in PHAs [149]. The lack of solvents and short analysis time reduces the possibility of harmful chemical exposure while offering a quick data output. However, because FTIR has less detection sensitivity and is ineffective at characterizing or detecting changes in PHA monomeric composition, it might be considered semiquantitative. It also cannot distinguish the difference between PHA mixes, copolymers, and heteropolyesters, either [98]. 

### 4.3. Chromatography

#### 4.3.1. Gas Chromatography–Mass Spectrometry (GC–MS)

Gas chromatography (GC) is the most widely used method for exact PHA determination as it is highly automated and accurate. It can give information about the exact monomeric composition of the polyester with high sensitivity. It requires the depolymerization of PHA into acids, diols, or esters prior to analysis. Braunegg et al. [150] reported the use of GC for the detection of PHB in bacterial cells. The cells were treated with a solution containing chloroform and methanol with concentrated sulfuric acid to induce methanolysis and were then subjected to GC for detection. Nowadays, this protocol is followed with some modifications to convert the PHA molecule into alkyl esters of PHA monomers which are then analyzed using GC. Bhuwal et al. [146] treated the PHB with sulphuric acid and methanol and then the methanolyzed PHBs were analyzed by GC–MS analysis. The molecular weight was found to be 256 kDa and 242 kDa for the two isolates. This method has paved the way for a large number of scl-PHAs and mcl-PHAs to be readily detected without the need for reference standards. Andler et al. [151] used GC–MS to evaluate their PHB sample isolated from *Bacillus cereus*, which was methanolyzed using chloroform and methanol/sulfuric acid (85:15 *v/v*) before it was injected. The inorganic phase was then put into GC–MS, which confirmed the polymer by revealing the presence of 3-hydroxybutyrate methyl ester molecules. GC–MS was used by Morya et al. [152] to characterize the monomeric structure of the recovered PHA from *Burkholderia* sp. The generated PHA contained 3-HB and 3-HV according to the fragmentation pattern analysis. Polyhydroxyalkanoates from *Cupriavidus* sp. ISTL7 were analyzed by GC–MS for which the lyophilized cells were methanolyzed at 100 °C followed by phase separation. It detected Fatty Acid Methyl Esters and 3-hydroxybutyrate, indicating that PHB was produced [136]. 

#### 4.3.2. High Performance Liquid Chromatography (HPLC)

HPLC is a chromatographic method that does not need numerous organic solvents and is superior to GC, which requires the methanolysis of PHA [153]. Kichise et al. [154] used HPLC to create an initial assay method for measuring cellular P(3HB) material. P(3HB)-accumulated cells were treated with hot concentrated sulfuric acid at 100 °C to convert P(3HB) to crotonic acid, which was followed by subjecting the sample through HPLC with an ultraviolet (UV) detector to test 210 nm absorption due to the presence of unsaturated crotonic acid bonds. A new method suggested by Satoh et al. [153] is based on alkaline digestion prior to HPLC. In this process 3-hydryoxybutyrate units were converted into 2-butenoate and 3-hydroxyvalerate units into 2-pentenoate using 2N NaOH and were then treated with sulfuric acid and analyzed using HPLC. HPLC necessitates the derivatization of polymer samples which is one of its drawbacks [142]. 

## 5. Recent Trends in PHA Applications

**Figure 4:**
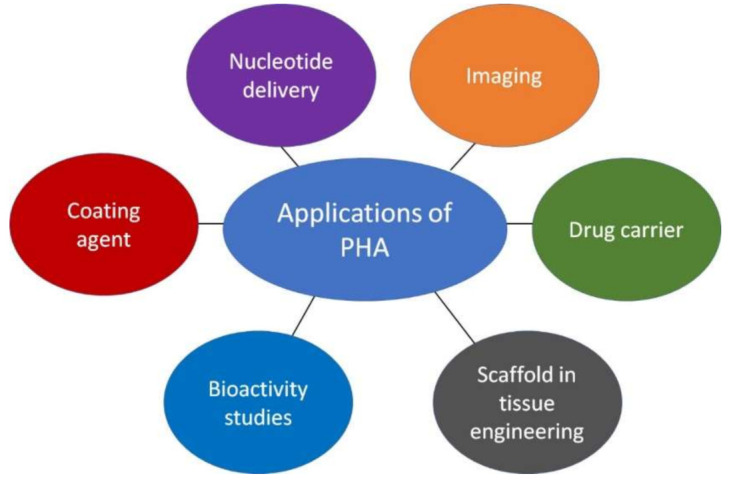
Applications of PHA.

PHAs differentiate themselves from other synthetic polymers on the market because they are ecologically benign and can cohabit happily with living tissues, making them a great resource [155]. They can be formulated and refined for use in agriculture, packaging, molding products, paper coatings, adhesives, films, and performance additives [156]. PHAs are a promising implant material as they are biodegradable, biocompatible, nontoxic, and tissue friendly with blood, bone, cartilage, and human cell lines as well as mammals [157]. Various therapeutics have been developed from PHAs such as sutures, articular cartilage repair devices and patches, cardiovascular patches, orthopedic pins, adhesion barriers, stents, guided tissue repair/regeneration devices, nerve guides, wound dressings, tendon repair devices, 3D custom-made bone marrow scaffolds, etc. as they lack cytotoxicity [158]. PHA has been shown to promote bone tissue growth as well [159]. Some of the important applications of PHA have been illustrated (Figure 4). In this review, the recent trends in applications of PHA are briefed as follows.

### 5.1. PHA as a Coating Agent

The packaging of food items with barrier coatings maintains moisture and oils within the package or lock in oxygen and moisture [160]. PHAs are an eminent biodegradable packaging material used as films, foils etc. [161]. They have properties similar to polypropylene and can be used for food wrapping [162]. The fact that they are water resistant makes them perfect for packaging materials such as, diapers, milk cartons, sanitary towels, razors, shampoo bottles, and disposable feminine hygiene products etc. [163]. PHB and PHBV provide antimicrobial effects onto the surface of antibiotic-loaded titanium (Ti) implants intended to prevent implant-associated infections by a gentamicin-controlled release phase. It was synthesized using the dip-coating technique and promised to prevent proliferation and biofilm formation [164]. A growing number of companies have been developing food packaging materials that combine traditional packaging materials with nanoparticles. They provide antimicrobial properties and improve mechanical performance too [160]. Castro-Mayorga and coworkers synthesized an antimicrobial polyhydroxyalkanoate that contained in situ-stabilized silver nanoparticles that inhibited the growth of *Salmonella enterica*. It was reported to offer an antimicrobial alternative to polyethylene for food packaging applications [165]. The paper industry also uses biopolymers as coatings to replace existing synthetic coatings. The high hydrophobicity of PHAs makes them insoluble in water and impervious to hydrolytic degradation. The thin, film-forming properties of PHA provide a protective barrier against oxygen and UV rays [166].

### 5.2. PHAs in Medical Devices

PHAs can be used for the development of surgical mesh, medical devices, orthopedic pins, surgical sutures, stents, repair patches, cardiac valves, staples, and screws as they are well tolerated by the immune system [167]. PHB can be used as microcapsules to encapsulate Langerhans cells, thereby restoring insulin synthesis and release, and as a material for cell and tablet packaging [168,169]. Paclitaxel, an anti-tumor drug, was electro spun with P(3HB-co-95 mol% 4HB) to examine drug release. The addition of paclitaxel increased the viscosity of the solution, resulting in a more stable jet during electrospinning. The experiment revealed the ability to entrap a drug into PHA, which may then be coated onto metal stents and be used as drug eluting stents [170].

### 5.3. PHAs in Agriculture

**Figure 5:**
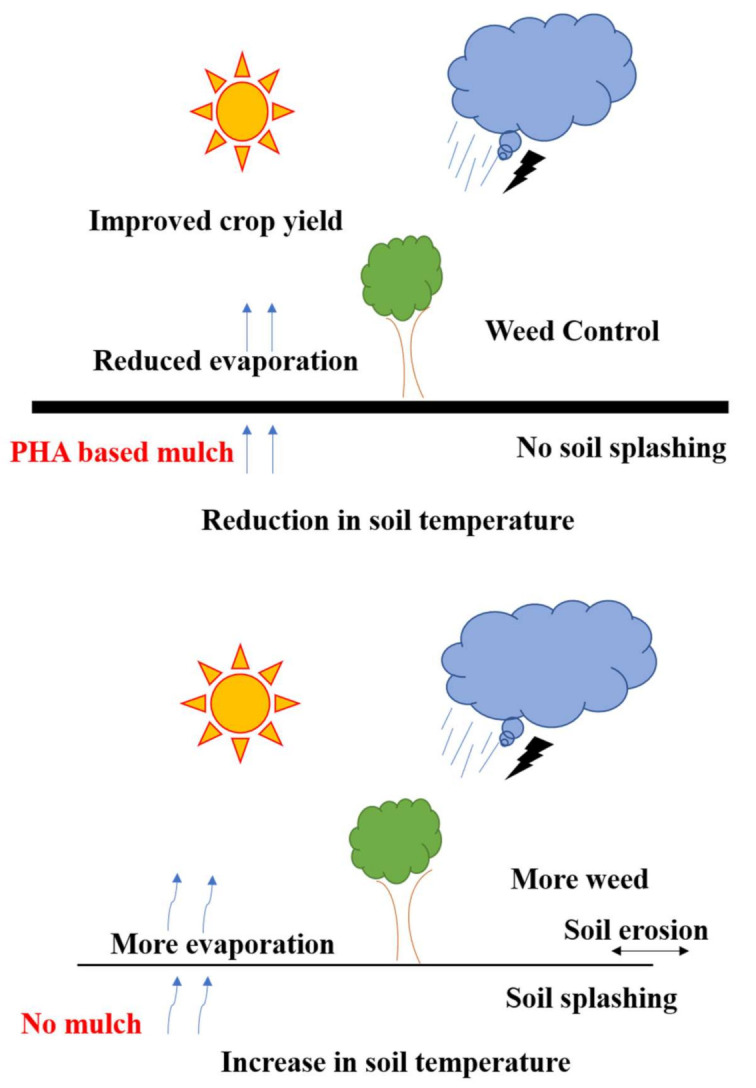
Effects of PHA-based mulching (Inspired from Sintim and Flury, [171]).

In recent years, easily degradable mulch has gained popularity, as old plastic (synthetic) mulch frequently ends up in landfills or is burnt, which in turn causes environmental pollution [171]. PHAs can be used to make soil friendly compostable greenhouse films, grow bags, and protection nets by replacing commercial plastics. Due to their excellent thermomechanical properties, PHAs can be used as environmentally friendly plastics in packaging industries too [172]. Mulch aids in better soil integrity, pollution control, moisture retention, and weed control (Figure 5). Biodegradable mulch has been designed based on a PHA copolymer of poly(3-hydroxybutyrate-co-3-hydroxyhexanoate) named Nodax™ and is patented by Danimer Scientific [173,174].

Agricultural nets made of biodegradable PHAs can be used to enhance crop growth, protect crops from natural climate fluctuations, insects, and birds, and avoid overheating, etc. It enables direct soil disposal, and, unlike plastic netting, it is easily compostable [175]. PHA based grow bags are in use nowadays as they do not tend to release harmful toxins upon degradation, unlike plastic growbags. They can act as a microbial growth matrix, can enable water denitrification, and are root friendly too [176].

## 6. PHAs in Nanotechnology

### 6.1. PHA Nanoparticles

An enhanced therapeutic value has been reported by the drug or bioactive compounds which are encapsulated in polymeric nanoparticles. This is due to the increased solubility, the increase in the proportion of drug released within the body, as well as the sustainability of the drug [177]. Based on their ability, the drugs repel or form a bond with the surface of the nanoparticles and perform a controlled drug release at a befitting time frame and position [178]. Polyhydroxybutyrate (PHB), a member of the PHA family of polymers produced from microbes, is feasible to administer in a diverse scope of significant applications due to its potential natural availability. PHB is also notable for its biodegradability, biocompatibility, and improved mechanical properties [179]. Polyhydroxybutyrate is used to activate the formation of tissues or organs and can conveniently be used in implantation medicine [180]. The human body does not make any immune response to the implant materials made with PHB [181]. These impressive properties of PHB make it an ideal material for nano-entrapment and the delivery of antimicrobial compounds to a target site [182]. Polyhydroxybutyrate (PHB) is widely used in the fields of medicine to make implants, in the food industry as a packing material, or in the production of nanoparticles to deliver a drug effectively at targeted sites without triggering any immunogenic reaction [183,184]. 

#### 6.1.1. Emulsion Solvent Diffusion

**Figure 6:**
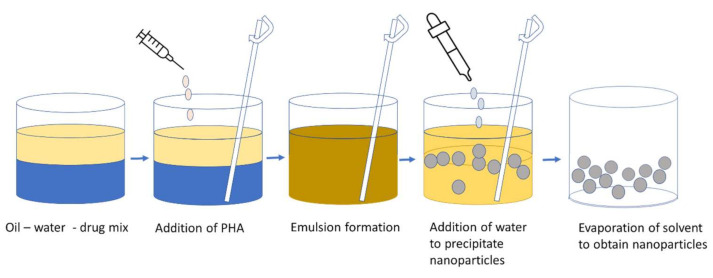
Emulsion Solvent Diffusion (Inspired from Kumar and Lal, [185]).

In the emulsion solvent diffusion method, PHA is mixed in an oil and water emulsion (sometimes drugs are added too) as the organic solvent is immiscible with water and an emulsion is formed upon stirring [185]. This emulsion is diluted by adding excess water under continuous mixing to overcome the miscibility of the organic solvent with water. Polymeric nanoparticles are formed which precipitate as the organic solvent diffuses into the water [186]. The emulsion evaporates to give nanoparticles (Figure 6). Guerrero et al. [187] suggested that nanoparticles are formed when the diffusion of solvent reaches supersaturation due to phase transformation. The size of the nanoparticles depends on the rate of stirring and the concentration of the polymer used. Sometimes sonication is also added to the step to produce nanoparticles of between 80 and 200 nm [188,189]. The emulsion solvent diffusion method has two types, namely, the single emulsion encapsulation method and the double emulsion encapsulation method. The prior method is for hydrophobic samples and the latter is for hydrophilic samples [190,191]. The type and number of surfactants such as polyvinyl alcohol (PVA), sodium dodecyl sulphate (SDS), and didodecyl dimethyl ammonium bromide (DMAB) can influence the size of the PHA nanoparticles [192]. A lower concentration of surfactant contributes to a high polydispersity and particle aggregation, whereas excessive surfactant can lead to a decrease in the drug loading efficiency. The oil-in-water emulsion–solvent evaporation process is the most common way to make PHA nanoparticles which involves the addition of an emulsifier, such as poly(vinyl alcohol), to an aqueous solution containing an organic phase, such as a PHA polymer dissolved in a solvent. The nanoparticles obtained by this procedure are between 100 and 200 nm in size and can be used as drug carriers [188,193].

#### 6.1.2. Nanoprecipitation

**Figure 7:**
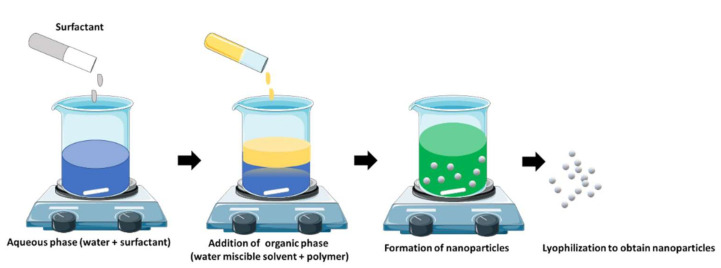
Nanoprecipitation method.

This involves a specific interaction between the organic solvent and the polymer to form a precipitate. The intermixing of the non-polar solvent with the aqueous portion of the solution that contains the polymer allows the formation of a nanoparticle and it can occur in the presence as well as in the absence of a surfactant [194] (Figure 7). This method is also known as the solvent displacement method [195,196]. Polylactic acids form characteristic nanospheres through the process of precipitation in the presence of a solvent whose polarity allows it to be miscible in all proportions when dissolved in water. The resulting mixture is injected into the water portion wherein the addition of a surfactant maintains the shape and stability of the nanocarrier [197]. The surfactant helps to create a stable shape with hollow cavities, where the encapsulation efficiency of the lipophilic drugs is increased [198,199]. Senthilkumar et al. [200] prepared PHB-based nanoparticles using SPAN 20 as a surfactant which were then loaded with a hydrophobic curcumin drug. The nanoparticles were found to be below 300 nm in size and spherical in shape.

#### 6.1.3. Dialysis

**Figure 8:**
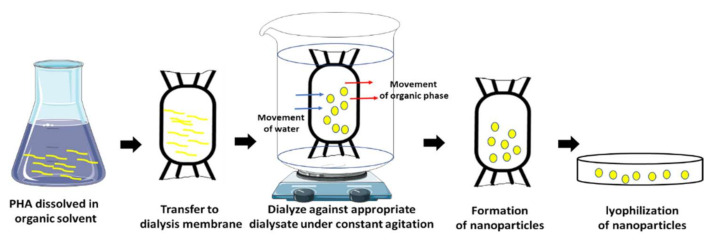
Dialysis method.

In the dialysis technique, the polymeric material is dissolved in an organic solvent and kept inside a dialysis tube. The choice of the pore size of the dialysis tube is highly crucial for the formation of polymeric nanoparticles [201,202]. Water enters the dialysis tube, and the hydrophobic polymers aggregate to form a colloidal nano/microsphere structure (Figure 8) where monomeric subunits or polymeric subunits can be used inside to make nanoparticles [50]. The solvent in which the polymer is dissolved, and the dialysate used plays a very important role when it comes to conserving the structure and maintaining a prolonged shape [201,203]. Spherical carvacrol-loaded polyhydroxybutyrate nanoparticles of 140 nm in size were also formed by the dialysis method which showed a uniform distribution. It showed a drug entrapment efficacy of 11% and released Carvacrol for up to 3 days [184].

### 6.2. Role of PHA in Fabricating Nanoparticles

#### 6.2.1. PHA as an Encapsulating Agent in Nanotechnology

**Figure 9:**
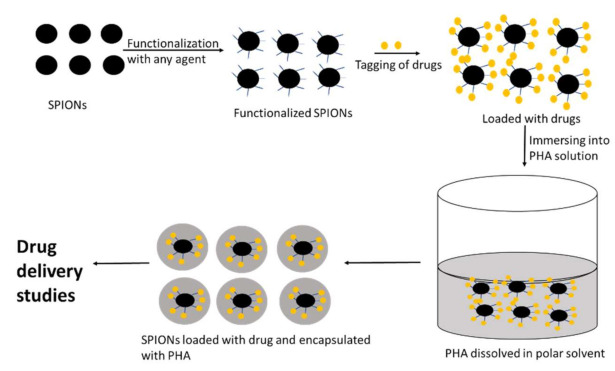
PHA-encapsulated SPIONs (Inspired from Samrot et al. [205]).

PHAs have desirable physical characteristics and excellent biocompatibility. As a result, they can be useful as coating agents for metal-based nanoparticles. Samrot et al. [204,205] utilized PHB for coating SPIONs which was functionalized with oleic acid and tagged with drug itraconazole as illustrated in Figure 9. It was observed that following encapsulation with PHB, the size of naked SPIONs increased from 40–45 nm to 100–130 nm. The drug-loaded nanoparticles had good drug release as well as inhibitory activity against *Pseudomonas aeruginosa* and *Candida albicans*. By entrapping (or wrapping to) bioactive nanoparticles through their biopolymeric chains, PHAs can stabilize metallic nanoparticles (ZnO-NPs, Ag-NPs) by acting as a framework for regulating the development of bioactive nanoparticles, thereby preventing aggregation [206].

#### 6.2.2. Phasin/PHA Synthase-Based Nanoparticle Synthesis

**Figure 10:**
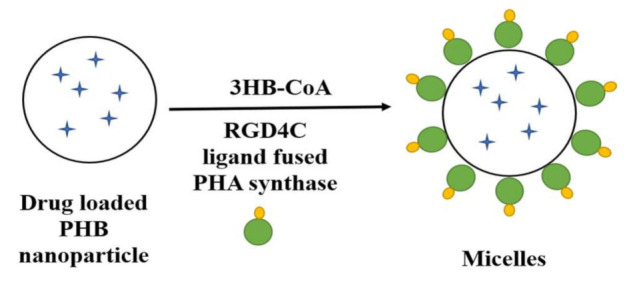
Synthesis of a PHA synthase-based nanoparticle (Inspired from Lee et al. [207]).

By utilizing PHA synthase in combination with ligands, PHA micelles can be produced. The PHA synthase converts 3-hydroxybutyryl-coenzyme A (3HB-CoA) to PHA by polymerizing it and keeping it covalently bound to specific amino acid residues within the enzyme [207]. A hydrophobic PHB nanoparticle loaded with the cancer inhibitory drug Nile red was prepared by coupling a PHB molecule with a RGD4C ligand using a PHA synthase enzyme. A protein–polymer shell of 46 nm in size was formed through oil-in-water emulsion solvent evaporation. It showed specific affinity to MDA-MB 231 breast cancer cells (Figure 10) [207]. Polyhydroxyalkanoate (PHA) synthase coupled to gold nanoparticles was found to accumulate PHB on its surface when 3-hydroxybutyrate-CoA was given as substrate, and it was stated that this PHB deposited gold nanoparticle can be used for cancer therapy [208].

#### 6.2.3. PHAs in Metal Nanoparticle Synthesis

Silver nanoparticles were produced by the chemical reduction of unpurified PHBV in a mixed culture fermentation medium containing fermented cheese whey. Crystalline and spherical NPs were produced and maintained for 40 days, suggesting that unpurified PHBV was an effective capping agent that prevented silver nanoparticle agglomeration and served as an agglomeration inhibitor. It also showed antibacterial activity against *Salmonella enterica*, a food borne microorganism [209]. Polyhydroxybutyrate-polylactic acid nanocomposites doped with copper and silver nanoparticles were synthesized. Both the PLA/PHB/Cu-NPs and PLA/PHB/Ag-NPs measured 93.24 nm and 123.71 nm in size, respectively, offering greater rigidity for packaging [210]. PHB–Ag nanocomposites were synthesized by Jayakumar et al. [211], which were found to be polydispersive and stable. They demonstrated significant antimicrobial resistance against common food pathogens such as *E. coli* and *Pseudomonas* sp. The PHB polymer retained its original structure after the addition of silver nanoparticles.

## 7. Characterization of Nanoparticles

The various elements of the nanoparticles that are synthesized, including particle size, morphological structure, functional groups, stability, crystallinity etc., and other characteristics, must be analyzed. The characterization can be completed through various techniques which are detailed below.

### 7.1. Fourier Transform Infrared Spectroscopy

Fourier Transform Infrared spectroscopy (FTIR) is an approach capable of retrieving an infrared spectrum of the transmission or absorption of a gas, liquid, or a solid sample [212]. When a photon is transferred by a molecule in the sample, it becomes excited and reaches a higher energy state from a lower energy state which results in vibrations in the form of either stretching, bending, twisting, rocking, wagging, and out-of-plane deformations occurring at varying wavenumbers (or frequencies). The absorbances of molecular vibrations under IR radiation are proportional to the abundance of the functional groups [213]. In PHB-based nanoparticles, peaks seen mostly at 1650.9, 1728, and 1725 cm^−1^ relate to a carboxyl group. The 1270 cm^−1^ peak represents the enol CO peak and the vibration for the benzoate-CH vibration shows around 850–960 cm^−1^ [5,214]. COC peaks are shown at around 1044 cm^−1^ [5]. FTIR-substantiated microbial biopolyesteric nanocarriers (MBPNc) loaded with amoxicillin and levofoxacin were created by Ojha and Das [215]. The carboxylic group of the MBPNc was represented by strong peaks at 3437, 3513, and 3531.66cm^−1^ in the spectra. When loaded with the drug, there was a shift in peaks or the formation of new peaks which confirmed the loading or conjugation of drug to the nanoparticle. In curcumin-loaded PHA-based nanoparticles, peaks around 1370–1350 cm^−1^ represent the C–O–H bond [5], whereas the peaks obtained between 1180 and 1160 cm^−1^ represent the C–O stretching of curcumin [216].

### 7.2. Scanning Electron Microscope (SEM)

Scanning electron microscope (SEM) is a type of electron microscopy which scans the sample with an attentive beam of electrons and produces images of the sample. The interaction between the electron and the atoms present in the sample generates various signals that enclose the data and provide predominant details regarding the sample’s surface, structure, and its composition [217]. A small quantity of the sample is processed and made accessible with a coating of gold or platinum sputtering to make it conductive. After that, the sample is observed through the electron microscope which displays the texture of the particles and shows if it is granular or crystal. It can also give details about the morphological structure and the pore diameter [218]. The image formation depends on the signals produced by the interaction between the electron beam and the sample. The primary electrons are made to fall on the sample, which then excite the specimen electron. The ionization of the sample atoms leads to the generation of secondary electrons. The secondary electrons are attracted towards the detector which converts the energy from the electron into visible light and is then amplified on the display screen [219]. Doxorubicin and sorafenib-loaded PHB nanoparticles were prepared and viewed under scanning electron microscopy and were found to be spherical in shape and 50 to 300 nm in size [220]. PHB was isolated from *Cupriavidus malaysiensis* USMAA1020 and was subjected to drug-loaded nanoparticle synthesis. Docetaxel-loaded P(3HB-co-4HB) nanoparticles were found to be spherical in shape with slight irregularities and were below 100 nm in size [221]. Senthilkumar et al. [5] produced a surfactant-less, spherical-shaped nanoparticle of around 250–350 nm in size, and the structure became bigger when loaded with curcumin. Nachiyar et al. [222] produced PHA nanoparticles of around 250–300 nm in size and they increased to 350–580 nm in size when loaded with levofloxacin. Thus, SEM analysis helps to determine the size and shape variation of the particles and also infers the difference between the drug loaded and unloaded nanoparticles. When Energy Dispersive X-ray Spectrometer (EDS/EDX) is associated with scanning electron microscope, qualitative and quantitative chemical analysis information of the sample can be obtained [223,224]. The EDX analysis of PHB/polyamine–NiO nanocomposites indicated the presence of Nikel, Carbon, and Oxygen where the ratio of Nickel: Oxygen was 7.33:31.08 [225].

### 7.3. Atomic Force Microscopy (AFM)

The particle size can be measured with ultra-high-resolution images captured with Atomic Force Microscopy (AFM). The atomic scale probe tip of AFM physically scans the samples at a sub-micron level [224,226]. A structural map of the sample is provided by this instrument and it is generated based on the forces acting between the surface of the particle and the probe tip. Contact and non-contact modes of scanning are the types of scanning in AFM analysis [227]. The probe taps the surface of the sample to generate a topographical map of the particle in contact mode and in non-contact mode, and the probe tip hovers on the conducting surface of the sample [228]. As the tip scans the surface of the sample, moving up and down on the surface of the sample, the laser beam is deflected from the cantilever. The difference between the light intensities between the lower and the upper photo detector from the deflected beam is recorded [229]. Even the non-conducting samples can be analyzed, and an image can be generated without any pre-treatment which enables the imaging of sensitive biological and polymeric nanostructures [230] such as PHA nanoparticles [214]. The most accurate size measurements of the particle and the size distribution of particles can be obtained from AFM analysis. This accuracy in the size measurement of particles makes an understanding of various biological conditions easier [231]. The AFM analysis was used by Senthilkumar et al. [214] to confirm the shape and structure of PHB nanoparticles. They were discovered to be smooth, round, but aggregated, with sizes ranging from 48 to 68 nm. In another study by Senthilkumar et al. [200, 214], AFM was used to distinguish the role of different surfactants and solvents on the PHB-based nanoparticle formation, where they produced nanoparticles ranging from 120 nm to 500 nm in size. PHB-b-PEG nanoparticles were reported to be around 200 nm in size [232].

### 7.4. Zeta Potential and Size Distribution

Zeta Potential can be defined as the electric charge on the surface of the nanoparticle. It is an important and widely used characterization method for size measurment in nanometers, which can be used for liquid samples. It can also be termed as “electrokinetic potential” [233]. This analysis determines the electric potential or surface charge of the sample in the diffuse layer. It can be used to study the stability and surface adsorption of the nanoparticles or even colloids [234]. The net surface charge of the nanoparticle is screened by a higher concentration of oppositely charged ions near the surface of the nanoparticle which leads to the formation of an “electric double layer”. The difference between the potential of the surface charge of the nanoparticle and the oppositely charged ions is called the Zeta Potential. The stability of the particle can be calculated by the magnitude of the Zeta Potential [235]. There will be either an increment or a decrement on the charge of the surface causing a higher or lower zeta potential [236]. In a study by Maia et al. [237], a change in the surface charges was found which was due to integration of the surfactant PVE onto the PHB–PHV (polyhydroxy valerate) microspheres and the zetapotential read between −8 mV and −18 mV. Another study by Francis et al. [238] showed that the PHB microspheres had negative zeta-potential values of −14.2 mV and −14 mV at pH 7 which was due to the higher concentration of PVA; this has been supported by many more studies [239]. However, the charge changed in accordance with different pH levels, as with a lower pH, there was a shift from a negative to a positive charge [240]. In an acidic pH < 4.0, C=O of PHB tend to be protonated resulting in a positive surface charge, whereas Lee et al. [241] found the zeta potential in acidic solutions to be a positive charge even when a higher concentration of PVA was used. A zeta size analyzer is very helpful where the size distribution can be easily determined. Sorafenib–doxorubicin-loaded PHB nanoparticles showed a mean average size of 199.3 ± 6.5 nm, were monodispersed, and showed a low polydispersity index of 0.071 ± 0.016 [220].

### 7.5. X-ray Diffraction Crystallography (X-RD)

X-ray diffraction is the most common characterization technique for PHA nanoparticles under submicron level [242]. It has the capacity to give details about purity, crystallinity, and morphology. The XRD pattern of PHB nanoparticles loaded with amoxicillin and levofoxacin showed peak values of 2θ = 13.5°, 19.35°, 22.88°, 25.37°, and 27.73°, corresponding to the (0 2 0), (1 1 0), (1 2 1), and (0 0 2) signals related to the XRD spectra of PHA [215]. Anbukarasu et al. [243] produced a thin film of PHB and subjected it to XRD where they obtained peaks at (020), (110), (021), (111), (121), (040), and (222), and their corresponding 2θ values were 13.5°, 16.85°, 19.8°, 21.4°, 25.5°, 27.2°, and 44°, respectively, representing orthorhombic crystal planes as PHB usually shows sharp peaks at 13° and 17° and an amorphic structure [244]. Thus, XRD enables us to study the structural changes of PHA-based nanoparticles too.

## 8. Applications of PHA Nanoparticles

Drug delivery systems have become an indispensable part of today’s medical care due to various benefits such as increased drug solubility and bioavailability, decreased toxicity, resulting in fewer potential side effects, and the ability to engineer the systems so that the successful targeting to specific cells or tissues is possible [245]. Anesthetics, antibiotics, anti-inflammatory agents, anticancer agents, antineoplastic agents, hormones, hydrogels, steroids, and vaccines have all been delivered using PHA-based nanoparticles (Table 2) [246,247]. Hydrophobic PHA nanoparticles can be modified to encapsulate hydrophobic drugs as well as hydrophilic drugs [248]. Curcumin, a hydrophobic compound, was successfully loaded onto PHA-based nanoparticles, which were smooth and spherically shaped with sizes ranging from 300 to 500 nm. The nanoparticles were discovered to continuously release drugs over a longer period [5]. Peng et al. [249] prepared hydrophilic insulin–phospholipid complex-loaded PHBHHx nanoparticles for regulated and sustained release to reduce insulin administration frequency. They showed a higher bioavailability when compared to the insulin solution. Poly (3 HV-co-3HB)-based nanoparticles were produced from PHA extracted from microalgae such as *Halomonas pacifica* ASL10 and *H. salifodiane* ASL11 showed antibacterial activity against *S. aureus* ATCC 25,923, and *P. aeruginosa* ATCC 27,853 [250]. Both the amoxicillin-loaded MBPNc (microbial biopolyesteric nanocarrier) and the levofoxacin-loaded MBPNc demonstrated a distinct zone of inhibition against *E. coli* at a concentration of 200 µL, with a diameter of 25 and 24 mm respectively [215]. The emulsification–diffusion technique was used to create PHA nanoparticles loaded with hydrophobic photosensitizer chemicals such as porphyrines and phthalocyanines. They were 169.0 to 211.2 nm in size and it was suggested that they may be employed as a hydrophobic photodynamic therapy in cancer treatment [251]. Nanoparticles made of amphiphilic PHA-mPEG copolymer nano carriers, loaded with thymoquinone, were spherically shaped and had a size of 112–162 nm. Studying the viability of core-shell nanoparticles in vitro demonstrated their compatibility with biological environments [252].

PHA nanoparticles have been utilized for enzyme immobilization as well. PHB extracted from *B. cereus* DV-4 was used for the synthesis of PHB nanoparticles by nanoprecipitation. PHB nanoparticles were immobilized with purified URAK enzyme, a fibrinolytic enzyme which had molecular weight of 46 kDa. PHB was dissolved in acetone before being injected into a buffer containing a purified URAK enzyme. Immobilization occurred due to the change in the solubility. Immobilization enhanced the activity of the URAK enzyme by 1.5 times [253]. 

An intein capable of self-cleaving that was pH-triggered has been used to bind target proteins from one side and bind PhaP from the other side. PHA nanoparticles can be attached to protein since PhaP has high affinity for attaching to them, thereby help in purifying the target protein [254].

**Table 2:** Different applications of PHA nanoparticles.

S No.	Polymer/Composite	Bioactivities	Drug Loaded with	Against What Type of Organism/Cells	References
1.	Poly[R]hydroxyalkanoate (PHA)	Antibacterial activity	Curcumin	*B. subtilis*	[5]
2.	Poly(3-hydroxybutyrate-co-3-hydroxyvalerate) (P (3HB-co-3HV)	Antibacterial Activity; Biocompatibility Test	Amoxicillin, Levofloxacin	*S. aureus, E. coli;* human embryonic kidney (HEK) cell line	[215]
3.	Poly(hydroxybutyrate-co-hydroxyhexanoate) (PHBHHx)	Hypoglycemic activity	Insulin	Diabetic rats	[249]
4.	Polyrhydroxyalkanoate (PHA)	Antibacterial activity	-	*S. aureus, P. aeruginosa*	[250]
5.	PHB, P(HB-12HV) and P(HB-50HV)	*In-vitro* photocytotoxicity	Porphyrines, Phthalocyanines	Human colon adenocarcinoma HT-29	[251]
6.	PHA–mPEG deblock copolymer	Biocompatibility Test	Thymoquinone	Prenatal rat neuronal hippocampal cells and NIH/3T3 fibroblast cell line	[252]
7.	Poly(3-hydroxybutyrate-co-3-hydroxyoctanoate)	*In vivo* anti-cancer activity	Doxorubicin	BALB/c nude mice	[255]
8.	Poly(ε-Caprolactone) and Poly(3-Hydroxybutyrate)	Anti-cancer activity	Chrysin	Human breast cancer cell line MDA-MB-231	[256]
9.	Polyhydroxybutyrate–Chitosan (PHB-Cs)	Antibacterial activity	Kaempferol	*Staphylococcus aureus, Acentibacter baumannii*	[257]
10.	Polyhydroxy butyrate/polyethylene glycol (Eu-Pi/PHB-PEG)	Anti-cancer activity	Eugenol, Piperine	Nasopharyngeal cancer (C666-1) cells	[258]

## Figures and Tables

**Table 1:** Synthesis of PHA from various microorganisms.

Microorganism	Carbon Source	PHA Yield (%w/v)	Solvent	PHA	Extraction Method	References
*Cupriavidus necator,*	Vegetable oil	95	Cyclohexanone	PHB	Solvent extraction	[52]
*Pseudomonas aeruginosa*	Castor oil and euphorbia oil	20–30	Chloroform	PHA	Solvent extraction	[53]
*E. coli* BL21	Glucose	20	Lysozyme and proteinase K	PHB	Enzyme extraction	[54]
*Sinorhizobium meliloti*	Sucrose	50	Triton X-100-EDTA	PHA	Surfactant–Chelate	[55]
*Bacillus cereus*	Glucose	9	Chloroform	PHB	Solvent extraction	[56]
*Pseudomonas oleovorans*	Octanoic acid	34	Chloroform	PH0-DEG	Solvent extraction	[57]
*Caulobacter crescentus*	Caulobacter medium, glucose	18	Chloroform	PHB	Solvent extraction	[58]
*Halomonas boliviensis*	Starch hydolysate, maltose, maltotetraose and maltohexaose	56	Chloroform	PHB	Solvent extraction	[59]
*Legionella pneumophila*	Nutrient broth		Chloroform	PHB	Solvent extraction	[60]
*Spirulina platensis*	Carbon dioxide	10	Methanol	PHB	Solvent extraction	[61]
*Rhodopseudomonas palustris* SP5212	Acetate, malate, fumarate, succinate, propionate, malonate, gluconate, butyrate, glycerol, citrate	7.7	Chloroform	PHB, PHBV	Solvent extraction	[62]
*Rhodopseudomonas palustris* SP5212	Hydroxybutyrate, valerat	49.06, 30	Chloroform	PHB, PHBV	Solvent extraction	[63]
*Aeromonas hydrophila*	Lauric acid, oleic acid	28	Hexane	PHA	Solvent extraction	[64]
*Agrobacterium tumefaciens* SU-11	Glucose	43	Chloroform and sodium hypochlorite solution	PHA	Solvent extraction	[65]
*Bacillus* sp.	Sucrose	11–41		PHB	Solvent extraction	[66]
*Escherichia coli*	Glycerol	5.2	Chloroform	PHA	Solvent extraction	[67]
*Serratia* sp.	Xylose	37.50	Methanol	PHB	Solvent extraction	[68]
*Enterobacter* sp. SU16	Glucose	40		PHA	Non solvent Precipitation	[69]
